# Neuroprotective effects of violacein in a model of inherited amyotrophic lateral sclerosis

**DOI:** 10.1038/s41598-022-06470-7

**Published:** 2022-03-15

**Authors:** Silvia Olivera-Bravo, Carmen Bolatto, Gabriel Otero Damianovich, Matías Stancov, Sofía Cerri, Paola Rodríguez, Daniela Boragno, Karina Hernández Mir, María Noel Cuitiño, Fernanda Larrambembere, Eugenia Isasi, Diego Alem, Lucía Canclini, Marta Marco, Danilo Davyt, Pablo Díaz-Amarilla

**Affiliations:** 1 Cell and Mol Neurobiol Lab, NCIC Department, Instituto de Investigaciones Biológicas Clemente Estable (IIBCE), Montevideo, Uruguay; 2 Histology and Embryology Department, Faculty of Medicine, Universidad de La República (UdelaR), Montevideo, Uruguay; 3 Genetic Department, IIBCE, Montevideo, Uruguay; 4 Tumoral Biol Area, Clin Biochem Department, Faculty of Chemistry, UdelaR, Montevideo, Uruguay; 5 Pharm Chem Lab, Organic Chemistry Department, Faculty of Chemistry, UdelaR, Montevideo, Uruguay

**Keywords:** Cell biology, Neuroscience

## Abstract

Amyotrophic lateral sclerosis (ALS) is a neurodegenerative disease characterized by progressive death of motor neurons and muscle atrophy, with defective neuron-glia interplay and emergence of aberrant glial phenotypes having a role in disease pathology. Here, we have studied if the pigment violacein with several reported protective/antiproliferative properties may control highly neurotoxic astrocytes (AbAs) obtained from spinal cord cultures of symptomatic hSOD1G93A rats, and if it could be neuroprotective in this ALS experimental model. At concentrations lower than those reported as protective, violacein selectively killed aberrant astrocytes. Treatment of hSOD1G93A rats with doses equivalent to the concentrations that killed AbAs caused a marginally significant delay in survival, partially preserved the body weight and soleus muscle mass and improved the integrity of the neuromuscular junction. Reduced motor neuron death and glial reactivity was also found and likely related to decreased inflammation and matrix metalloproteinase-2 and -9. Thus, in spite that new experimental designs aimed at extending the lifespan of hSOD1G93A rats are needed, improvements observed upon violacein treatment suggest a significant therapeutic potential that deserves further studies.

## Introduction

Amyotrophic lateral sclerosis (ALS) is a neurodegenerative disease characterized by progressive loss of motor neurons and paralysis that becomes lethal within 1–5 years from diagnosis^1–3^. Rapid evolution and the younger age of affected patients compared to Alzheimer´s and Parkinson´s diseases^3^ make ALS of considerable medical and social attention despite its low incidence (1–2.6:100,000)^1–3^. ALS is mostly a sporadic disease that results from yet unknown interactions among the environment, genes and age^2^. However, a minor percentage of cases is linked to inheritable genetic abnormalities in which mutations in the enzyme Cu/Zn superoxide dismutase-1 (SOD1) seem responsible for up to 20% of the familial types and 1% of the total cases^2–4^. Interestingly, when some of the SOD1 mutations identified in ALS patients were expressed in mice and rats, it elicited the most relevant pathognomonic signs of the disease including motor neuron death, glial reactivity and progressive paralysis^5^. In addition, data obtained from hSOD1G93A models showed that motor neuron death only occurs if the mutated enzyme is simultaneously expressed in motor neurons and in astrocytes or microglial cells^6–8^. Furthermore, astrocytes obtained from patients of sporadic and familial motor neuron diseases were neurotoxic^9,10^, even when SOD1 is not involved^10^. Therefore, neurotoxic astrocyte phenotypes may greatly contribute to motor neuron death in ALS.

Recent evidence showed that a subset of reactive/aberrant astrocytes were isolated from the spinal cord of hSOD1G93A murine models because of their proliferative potential that allowed an oligoclonal expansion without replicative senescence^11^. Isolated astrocytes were particularly neurotoxic^11,12^, either by gain of yet unknown deleterious properties^11^ or by loss of homeostatic functions such as lack of expression of glutamate transporters^11,12^. This subgroup of neurotoxic astrocytes, that was called AbAs (as the acronymum of aberrant astrocytes), clearly presented a phenotype that was distinctive from homeostatic astrocytes^11^, expressed markers of undifferentiated astrocytes such as high levels of S100β and connexin  43, low expression of glial fibrillary acidic protein (GFAP)^11^, absence of gliofilaments and abundance of microtubules^14^. AbAs proliferated faster than neonatal or adult astrocytes, they could replicate more than 20 times preserving their exceptional selective toxicity to motor neurons^11,13^, and showed permanent absence of contact inhibition that allowed them to grow in invasive tridimensional aggregates enriched in extracellular matrix^14^. Previous reports also provided powerful evidence about AbAs disturbances in cellular communication, autophagy and proteostasis as well as on their exacerbated endoplasmic reticulum (ER) stress and disrupted lipid metabolism^13,14^. Moreover, likely due to their significant mitochondrial dysfunction^14,16^, AbAs experienced the Warburg effect^15^ described in cancer cells since the glycolytic metabolism was their main energetic source instead of pyruvate mitochondrial oxidation^16^. Thus, AbAs and cancer cells might share similar features including some common energy production pathways as well as increased cell proliferation rate and absence of replicative senescence. Therefore, we have proposed that drugs that inhibit cancer cell proliferation, and ideally exhibit multifunctional beneficial properties, may not only control AbAs, but also may show protective effects on ALS models.

The bisilindole pigment violacein (3-[1,2-dihydro-5-(5-hydroxy-1H-indol-3-yl)-2-oxo-3H-pyrrol-3-ylidene]-1,3-dihydro-2H-indol-2-one) is produced as a secondary metabolite by diverse bacterial strains, including the *Janthinobacterium sp* UV13 that grows in the Antarctic glaciers^17^. Violacein is a broad-spectrum bioactive compound with demonstrated anti-microbe, immunomodulatory and antioxidant properties^18–22^. In addition, several reports showed that violacein has anti-tumor properties on many cancer cell lines and types^18,20,21,23–26^ in a wide range of concentrations (submicromolar to micromolar)^17,26,27^, although the causes of differential cell susceptibility remain unknown.

The proposed mechanisms for violacein actions include the increase in reactive oxygen species, the activation of caspases or of mitochondrial and non-mitochondrial apoptotic pathways^26^. Other authors report that phosphorylation of the p38 mitogen-activated protein kinase or up regulation of the nuclear factor kappa-B (NFκB) pathway may have a role in the control of cancer cells^23–25,28^. Antioxidant properties^19^ or immunomodulatory activities seen in animal models could also underlie violacein effects^18,22,29,30^. Violacein actions linked to inflammation include the maintaining of  the balance between pro- and anti-inflammatory cytokines^22^ and the modulation of  tumor necrosis factor alpha (TNF-α)^22,31^ or interleukin (IL) 6^30^ levels. Interestingly, some of these actions include the inhibition of the proteolytic activity of matrix metalloproteinase (MMP) -2 and downregulation of the interactions that control cell migration and invasion in breast cancer cell lines^31^. In addition, MMP-2 and -9 can potentiate inflammatory pathways by converting inactive cytokines into their biologically active forms by either cleaving their membrane-attachments or their inactive zymogens^31–33^. In turn, pro-inflammatory cytokines may induce the conversion of catalytically inactive proMMP-2 and -9 into their active forms^31–33^, thus chronically sustaining and potentiating inflammatory cascades^32,33^.

It has been reported that MMP-2 and -9 increased expression along ALS progression^31–33^ and MMP-9 has been proposed as a determinant of selective motor neuron vulnerability^33^. In addition, there is strong evidence about  the involvement of both MMPs in ALS pathology either through direct neurotoxic effects or indirectly by eliciting cell death upon exacerbated degradation of extracellular matrix proteins^32^ or by triggering glial cell activation and disturbing their homeostatic signaling^31–33^. Glial reactivity increases and sustains neuroinflammation^3,6–8,10^. In turn, chronic neuroinflammation^8,11,31–33^ may promote the emergence of reactive neurotoxic glial phenotypes that release pro-inflammatory molecules amplifying and perpetuating CNS damaging cascades^11,34^. Violacein could disrupt this deleterious feedback through different mechanisms and targets. On one hand, it can inhibit inflammatory cascades^18,22,29,30^ likely abrogating some pathological responses such as the appearance of aberrant glial phenotypes. On the other hand, anti-proliferative properties and differential cytotoxicity^23–25,28^ may allow the selective control of the emergent neurotoxic phenotypes that share common features with cancer cells as has been proposed for some CNS neurotoxic cells. Thus, the violacein protective actions could extend to the CNS.

To validate this hypothesis, we have proposed that violacein could selectively control the aberrant glial cells named as AbAs that emerged during disease progression^11,14^ in the rat ALS model hSOD1G93A^5^, and also have tested if the treatment of these animals with violacein may result in protective effects.

## Methods

All methods were performed in accordance with the relevant guidelines and regulations. Table 1 shows the number of animals, independent experiments and replicates done.

**Table 1:** Number of animals, experiments and replicates performed.

	Independent experiments		Replicates per experiment	
**a) Approaches in cell cultures**				
AbAs	6 Tg terminal rats		1, serial passages	
AA	6 age-matched Non-Tg rats		1, up to 3 passages	
Viability (SRB)	7		5	
Migration and proliferation	5		5–7	
Functional analysis (MTT, JC1, C-DCF, MCB)	5 in each parameter		5–9	
Immunocytochemistry	6		3	

Procedure, age and independent experiments	Tg rats		Non-Tg rats	
	Treated	Untreated	Treated	Untreated
**b) Animals**				
Injection (150 day-old) (N = 3)	18	18	18	18
Processing (~ 190–200 day-old)				
for biochemical assays	12	12	12	12
PFA fixation (~ 190–200 day-old)	6	6	6	6

	Tg rats		Non-Tg rats	
**c) Approaches performed in tissue samples: animal numbers and replicates in parenthesis**				
Muscle histology	7 (5)	7	7	7
Muscle zymography	12 soleus (5)	12 soleus	12 soleus	12 soleus
Spinal cord zymography	12 (7)	12	12	12
NMJ structure	12 soleus (5)	12 soleus	12 soleus	12 soleus
Spinal cord Nissl staining	6 (5)	6	6	6
Immunofluorescence	6 (5)	6	6	6
Dot blots	7 (3)	7	7	7

Different sections show: a) approaches performed in vitro and b) animals employed, and c) approaches done in tissue sections or homogenates with the number of animal samples employed in each one and replicates in parenthesis. Abbreviations: *C-DCF* carboxy-dichlorofluorescein, *MCB* monochlorobimane, *MTT* 3-(4,5-dimethylthiazol-2-yl)-2,5-diphenyltetrazolium-bromide, *NMJ* neuromuscular junction, *Tg* Transgenic (hSOD1G93A) rats, *Non-Tg* Non transgenic rats, *PFA* paraformaldehyde.

### Animals

Hemizygous NTac:SD-Tg(SOD1G93A)L26H male rats^5^ (Taconic) (Tg) and non-transgenic brothers (Non-Tg) were used (12 h light/dark, 22 ± 1 °C, water and food ad libitum). Animal care and experimental protocols (N° 004/09/2015 and 001/11/2021) were approved by the IIBCE Ethical Committee for the Use of Animals (CEUA-IIBCE) that follows the No 18611 Uruguayan Law for Care and Use of Laboratory Animals.

### Cell cultures

AbAs: lumbar spinal cord homogenates from ~ 200 day-old Tg males were seeded until confluence^11^, passaged 4 times to eliminate contaminant cells and passaged 5–10 times to propagate AbAs^11,14^.

Adult astrocytes (AA): lumbar spinal cords from ~ 200 day-old Non-Tg males were processed as in neonatal astrocyte cultures^11,14,35^.

Rat glioma C6 cells^36^ expressing astrocyte markers were selected as positive controls of the anti-tumor properties of the purified violacein used in this work^18,20,21,23^ because of their shared lineage and previous comparative results with AbAs and astrocytes^11^.

Medium (DMEM + 10%FBS) and conditions were maintained in all cell cultures^11,35^.

### Violacein log P calculation

Ability to cross the blood brain barrier was estimated according to OECD guides^37–39^. Briefly, standard’s RP-HPLC running conditions were optimized until reaching a linear correlation between log K [retention coefficient: log (stationary phase concentration/mobile phase concentration)] and log P [partition coefficient: log (concentration_octanol_/concentration_water_)]. Then, violacein^17^ was ran under the optimized conditions, log K determined and log P interpolated into the standard curve (Table 2).

**Table 2:** Determination of log P for violacein.

Standards	Tr (min)	(Tr-T0)/T0	Log P	Log K
Uracil (T0)	1.687			
4-aceyilpiridine	2.059	0.220509781	0.5	− 0.656572143
Acetanilide	2.367	0.403082395	1	− 0.39460617
Nitrobenzene	3.503	1.076467101	1.9	0.032000762
Bromobenzeno	7.785	3.614700652	3	0.558072337
Bencilbenzoate	12.285	6.282157676	4	0.798108833

	Tr (min)	(Tr − T0)/T0	Log K	Log P
**log K = 0.4258 logP – 0.8183; R**^**2**^** = 0.9857**				
Violacein	3.994	1.367516301	0.135932512	2.241034551
ND 56	7.01	3.155305276	0.499041384	3.093803155
ND 70	4.163	1.467694132	0.166635558	2.313141282
ND 80	3.926	1.327208062	0.122939011	2.210519049

Uracil, 4-acetylpyridine, acetanilide, nitrobenzene, bromobenzene and benzyl benzoate, used as standards, were loaded in a C18 reverse phase Eclipse Plus column (5 µm, 4.6 × 150 mm) with 30:70 water:methanol as mobile phase, run at 1 ml/min flow, and detected at 220 and 254 nm using a diode array detector. All experiments were done in triplicate and results expressed as the arithmetic mean allowing calculating log K for each standard loaded. Then, standard´s Log K versus log P was graphed and linear regression done requiring R^2^ ≥ 0.98. After that, violacein was loaded and ran under optimized conditions. Violacein log K was determined from the run and log P calculated from the standard chart.

### Violacein effects on cell viability

Viability of confluent cultures^11,16,35^ exposed to vehicle (DMSO 0.25%) or 0–1,500 nM violacein (24 h) was assessed with sulforhodamine B (SRB)^17^. Briefly, cell proteins were precipitated (50% trichloroacetic acid, 30 µl/well, 1 h, 4 °C); labelled (0.4% SRB, 45 min, RT), dissolved (100 mM Tris base, 100 µl) and specific optical density (OD) (530–690 nm) measured and related to vehicle.

### Violacein effects on cell migration and proliferation

AA or AbAs (30,000 cells/200 µl) seeded on wells containing cell stoppers during 48 h, were imaged before and after treatment (0.25% DMSO or 0–200 nM violacein, 48 h). Initial and final cell free areas (CFA) were measured; cell covered area calculated [100*(CFA_i_-CFA_f_)/CFA_i_] and related to vehicle conditions.

Simultaneously to violacein treatment, 25 µM of 2-bromodeoxyuridine (BrdU) was added to each well to assess cell proliferation^35^.

### Violacein effects on functional parameters

Analysis were made in confluent AA or p6 AbAs treated during 6 h^40^ (0.25% DMSO or 0–200 nM violacein). All parameters, except JC1, were related to 1 µg/ml Hoechst 33342 fluorescence (excitation: 360; emission: 405 nm) simultaneously determined in 3 wells per experimental condition^40^. In all cases, values were related to vehicle conditions.

Mitochondrial NAD(P)H activity was assessed by 3-(4,5-dimethylthiazol-2-yl)-2,5-diphenyltetrazolium-bromide (MTT) reduction^41,42^. Briefly, 0.1 mg/ml MTT was added (45 min, 37 °C), medium replaced (100 µl DMSO), OD (570 and 630 nm) measured and (OD_570nm_-OD_630 nm_)/Hoechst fluorescence ratios determined.

Mitochondrial potential was evaluated with the ratiometric probe JC1 (excitation: 488 nm; emissions: 520 and 590 nm)^40^. Briefly, JC1 was added to the cultures (3 µM, 15 min, culture oven), rinsed and fluorescence measured as 590 nm/520 nm ratio.

Intracellular reactive oxygen species were evaluated by carboxy-dichlorofluorescein fluorescence upon incubating cells with 6-carboxy-2′,7′-dichlorodihydrofluorescein-diacetate (5 µM, 45 min, 37 °C)^40^ and measuring at 520 nm/485 nm ratio.

Glutathione levels were estimated by measuring fluorescent monochlorobimane-glutathione adducts^40,43^ formed upon adding monochlorobimane to cell cultures (30 µM, 45 min, 37 °C). After disrupting cell membranes (0.1% Igepal), the 460 nm/395 nm fluorescence ratio was determined.

### Animal treatment and monitoring

150 day-old Non-Tg and Tg^5^ males received a weekly intraperitoneal injection (300 µl) of violacein (300 nmole/kg)^17^ or vehicle (0.5% DMSO) until ~ 200 day-old. The dose was chosen because of the AbAs selective vulnerability over AA. A weekly administration was selected over two or three injections because of less animal handling and similar protective results.

Body weight was measured weekly up to 170 day-old and then every 2 days^5^. Body weight peak and first abnormal gait determined disease onset in Tg rats^5,16^.

Disease progression was ranked by comparing Tg with age-matched Non-Tg animals as follows: (5) freely moving; (4) abnormal gait; (3) a leg paralyzed; (2) both legs paralyzed; (1) end stage (inability to recover right position when on their back)^16^.

### Animal processing

At ~ 190–200 day-old, when a Tg—untreated rat reached the end stage, animals of each experimental condition were anesthetized (90:10 mg/Kg ketamine:xylazine)^16^, guillotined, lumbar spinal cord and soleus muscles dissected and weighted. A half of each sample was fixed by immersion in 4% (spinal cord) or 0.5% (soleus) PFA (24 h, 4 °C) and submitted to morphological analysis. The other half was collected in lysis buffer^44^, sonicated, spun, protein concentration determined (Bicinchoninic acid method) and frozen (− 80 °C) until analyzed by zymography or dot blot^44^. Some animals were transcardially perfused with 4% PFA^45^.

### Muscle histology

Fixed muscles were divided in halves. Each half was dehydrated, paraffinized^46^ and the central portion cut in 6 μm transverse sections that were stick to slides. Dried sections were deparaffinized^46^ and stained with H&E and Masson or Cajal-Gallego trichromes^46^. Number and areas of entire fibers, collagen and muscle were determined using FIJI^47^.

### Zymography

Spinal cord and muscle homogenates (30 µg protein) or recombinant MMP-2 and MMP-9 (positive controls) were diluted in sample buffer (0.08 M, pH 6.8 Tris base + 0.017% SDS + 5% glycerol + 0.02 mg/ml bromophenol blue), seeded on 10%:0.1% polyacrylamide:gelatin gel and electrophoresed (100 V)^44^. Gels were washed (2.5% Triton X-100), incubated (50 mM, pH 7.6 Tris buffer + 5 mM CaCl_2_ + 20 mM NaCl + 0.005% Brij 35, 18 h, 37 °C), stained (1% Coomassie Brilliant Blue R-250) and distained (40%:10% methanol:acetic acid) until detecting gelatinolytic white bands^44^. Gels were scanned in an iBright FL1500 Imaging System. Positive bands were measured with FIJI Gel analysis tool.

### Immunofluorescence generalities

Unless stated, these steps were done in all cases: permeabilization (0.1% Triton X-100, 30 min, RT); un-specific binding blockade (60 min, PBS:5%BSA); primary antibody incubation (overnight, 4 °C, wet close chamber); secondary antibody incubation: (1:1000 anti-mouse or anti-rabbit Alexa Fluor 488 or 543; 90 min, RT) and mounting (50% glycerol:1 µg/ml Hoechst 33342). Positive and negative controls were done for each antibody employed^11,14,16,35,40^.

Immunocytochemistry^11,14,16,40^: Cells were fixed (4% PFA, 20 min, RT) and then immunofluorescence performed. Antibodies employed were: 1:600 anti-S100β (S2532, Sigma), 1:400 anti-GFAP (G9269, Sigma) or 1:300 anti-Iba1 (ab178846, abcam). In some cell experiments, Alexa Fluor 488 Phalloidin (A12379, Invitrogen) (1:200, 20 min) incubation followed the secondary antibody incubation. Then, cells were rinsed and mounted.

BrdU immunolabeling to assess cell proliferation^35^: DNA was denatured (2 N HCl, 45 min, RT), HCl washed (6 × 10 min, PBS) and immunofluorescence performed using an anti-BrdU antibody (1:1000, MO-744, Dako).

Muscle MMP-2 and -9 immunoreactivity: Deparafinized^46^ muscle sections were retrieved (20 µg/ml proteinase K, 5 min, 37 °C)^45^ and submitted to immunofluorescence using anti-MMP-2 (1:300, #436000, Invitrogen) or anti-MMP-9 (1:300, #PA5-13199, Invitrogen) primary antibodies.

Spinal cord immunofluorescences: 30 µm vibratome sections were assayed as stated above using the following primary antibodies: i- anti-MMP-2 or -9 (1:300); ii- anti-S100β (1:600), anti-GFAP (1:400) or anti-Iba1 (1:300), to assess glial reactivity; iii- anti-MMP-2 (1:300)/SMI32 (1:800, #SMI32-P, Covance)/Tomato lectin (1:300, L0651, Sigma-Aldrich) to assess signal co-localization; iv- anti-IL-1β (1:1000, ab234437, abcam or 500-P21BG, Peprotech) or anti-TNF-α (1:500, ab6671, abcam or 500-M26, Peprotech) to assess inflammation^11,17^.

NMJ recognition and analysis: teased muscle fibers were labelled as stated in Bolatto, Olivera-Bravo et al. (2021)^47^. Probes and antibodies employed were biotin-αBungarotoxin (1:500, B1196, Invitrogen), SMI31 (1:800, #SMI31-P, Covance), anti-mouse Alexa Fluor 488 (1:1000), and streptavidin-Alexa Fluor 555 (1:1000). NMJ parameters were measured and calculated as previously reported^47–49^.

### Nissl staining

Thirty µm spinal cord sections mounted on gelatin-covered slides were dried, immersed in 1:1 ethanol:chloroform (overnight, RT)^46^, re-hydrated^46^, and exposed to 0.1% cresyl violet (20 min, 37 °C). Then, sections were differentiated, dehydrated, and mounted in DPX^46^. Density of motor neurons and of small nuclei were determined in the IX Rexed lamina^11^.

### Imaging

Light images of muscle histology and spinal cord Nissl stainings were obtained with a Nikon Eclipse E400 microscope. Immunofluorescences were imaged as Z-stacks at 1024 × 1024 or 2048 × 2048 in laser scanning confocal microscopes (Olympus FV300 or Zeiss470). Constant acquisition parameters were determined with negative and positive controls to avoid saturation or false signals. Signal intensity was measured using the mean gray value parameter (MGV, FIJI). Five independent persons made quantitative analysis.

### Dot blots

A 1 cm nitrocellulose membrane strip was spotted with spinal cord homogenates (30 µg protein), dried, blocked (5% BSA/TBS-T, 1 h, RT) and incubated (30 min, RT) with the following antibodies: anti-IL-1β (1:1000), anti-TNF-α (1:1000), anti-IL6 (1:500, ab229381, abcam) or anti-β actin (1:2500, A5441, Sigma). Then washed 3x (TBST-T) and incubated with HRP-conjugated secondary antibodies (1:2000, 30 min, RT)^50^. BSA and recombinant cytokines were used as negative and positive controls, respectively. Signals were developed (ECL, Thermo Fisher, 34577) and read (iBright FL1500 Imaging System). Integrated density was measured using FIJI.

### Statistics

Data were expressed as mean ± SEM. Statistical tests used from GraphPad Prism 8.4 were ordinary one-way ANOVA and Tukey’s post-hoc comparisons under normality, non-parametric Kruskal–Wallis test when normality failed and Long-rank Mantel Cox test for survival analysis. Statistical signification was determined at p < 0.05.

### Methodology statement

All authors confirm that all methods described in this manuscript were performed in accordance with ARRIVE guidelines.

## Results

### Aberrant astrocytes (AbAs) were selectively vulnerable to purified violacein

**Figure 1:**
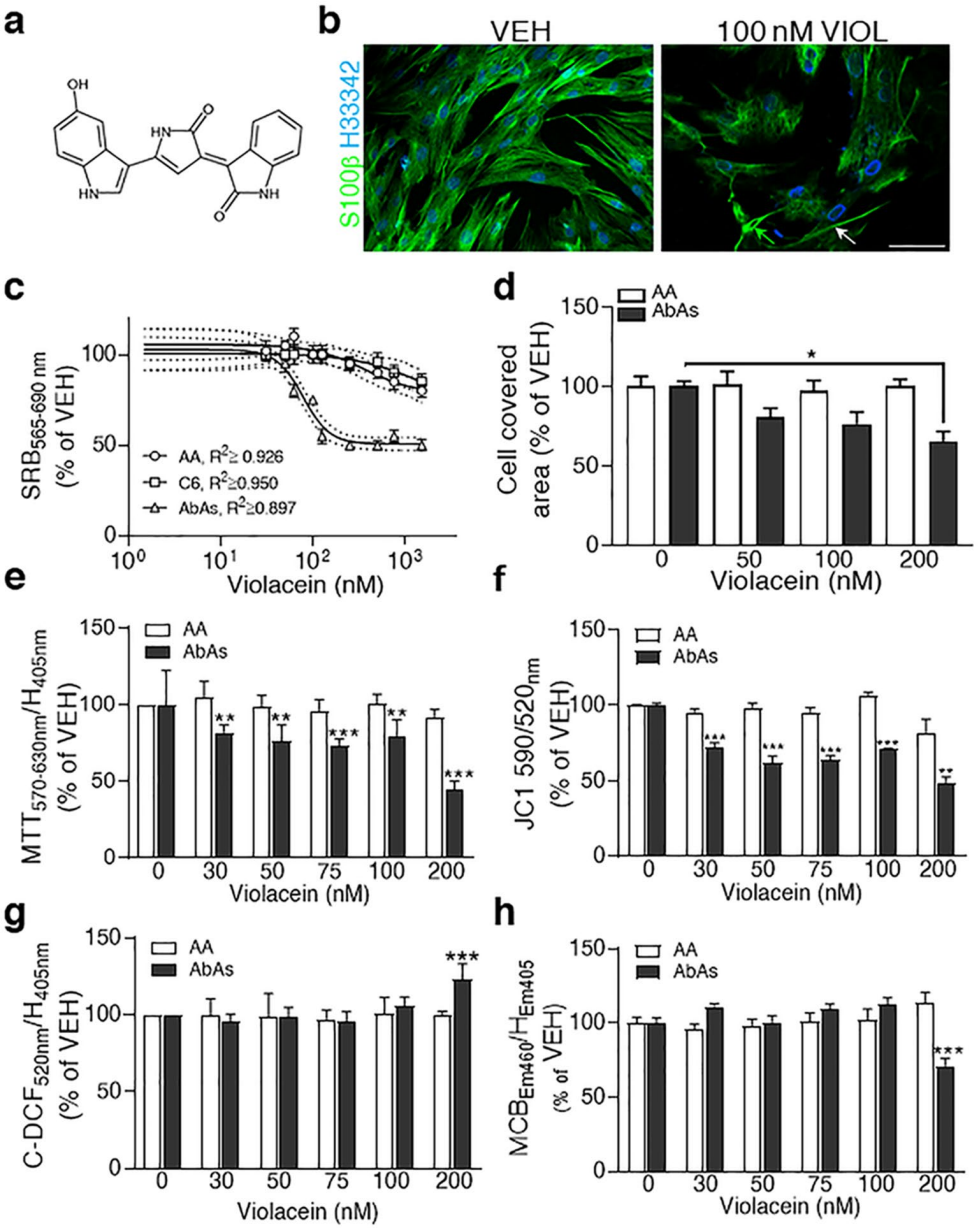
Violacein selectively controlled AbAs viability and decreased their antioxidant defenses. (**a**) Violacein molecular structure. (**b**) Representative confocal images of p6 AbAs showing a fibrilar S100β staining (green) upon a 24 h treatment with 0.25% DMSO (VEH) or 100 nM violacein. Violacein reduced cellular density and modified S100β appearance showing more condensed cytoskeleton (green arrow) or nuclear envelope and altered substrate adhesion (white arrow). Hoechst 33342 stained cell nuclei (blue). Calibration: 50 µm. (**c**) Concentration-inhibition curves in confluent p6 AbAs (△), AA (○) or C6 (□) cells cultures treated with 0–1500 nM violacein (24 h, sulforhodamine B (SRB) assay). Solid lines represent the best fittings whereas dotted ones indicate 95% confidence intervals. (**d**) Violacein effects on AA and p6 AbAs migration assessed by percent of cell covered area upon 48 h of treatment with vehicle or 0–200 nM violacein. At 200 nM, violacein reduced AbAs migration when compared with vehicle (p = 0.0181). (**e**) Violacein inhibitory effects on AbAs mitochondrial NAD(P)H activity evaluated by MTT reduction related to Hoechst 33342 fluorescence. Reduction was found since 50 nM violacein compared with vehicle (p = 0.0334) or with AA submitted to the same concentration (p = 0.004). Hoechst 33342 was abbreviated as H. (f) Selective action of violacein on AbAs mitochondrial potential assessed with JC1. Decreases in AbAs were found at 30 nM (p = 0.0005) and higher concentrations. At 200 nM, violacein marginally decreased AA mitochondrial potential (p = 0.057). (**g**) Effects of violacein on levels of oxidative stress in AA and p6 AbAs assessed by carboxy-dichlorofluorescein (C-DCF) fluorescence and expressed as C-DCF/Hoechst 33342 fluorescence ratios related to vehicle. At 200 nM, violacein increased C-DCF relative emission in AbAs when compared with vehicle (p = 0.0047) or with AA (p = 0.001). (**h**) Effects of violacein on total glutathione levels were seen at 200 nM with reduced formation of glutathione-monochlorobimane (MCB) adducts in AbAs respect to the vehicle (p = 0.0007) but not in AA. Experiments and replicates appear in Table 1 for all of the figures. (**d**–**g**) symbols: □: AA; ■: AbAs.

Violacein purified from *Janthinobacterium *sp. UV13 (Fig. 1a) selectively affected the viability of AbAs (Fig. 1b and S1a–c) when compared with confluent cultures of Non-Tg adult astrocytes (AA, Fig. 1c and S1d) or with C6 rat glioma cells (Fig. 1c). The estimated IC_50_ value for AbAs was ~ 175 nM whereas for AA and C6 cells it exceeded 1000 nM. In addition, AbAs viability in response to violacein did not differ between 150 and 300 nM for 24 h of treatment. Therefore, we have selected the range of 0–200 nM to analyze violacein effects on some functional parameters.

Upon 48 h of incubation, 200 nM violacein, but not lower concentrations, impaired AbAs migration when compared with vehicle condition (Fig. 1d; ~ 30 ± 10%; p = 0.0181). Violacein also decreased AbAs proliferation (~ 25% at concentrations higher than 30 nM, p = 0.0015), NAD(P)H oxidoreductase activity (~ 24% at concentrations higher than 50 nM, p = 0.0334) (Fig. 1e) and mitochondrial potential (~ 28% since 30 nM and higher; p = 0.0005) (Fig. 1f). At 200 nM violacein, mitochondrial potential of AA was marginally affected (p = 0.057) (Fig. 1f). Once exposed to 200 nM violacein, but not at lower concentrations, AbAs increased the levels of ROS (~ 23%; p = 0.0047) (Fig. 1g) and decreased total glutathione levels (~ 29%; p = 0.0007) (Fig. 1h). Confluent cultures of AA did not show significant alterations in both parameters analyzed once exposed to violacein.

### Log P value suggests that violacein might cross the blood brain barrier

**Figure 2:**
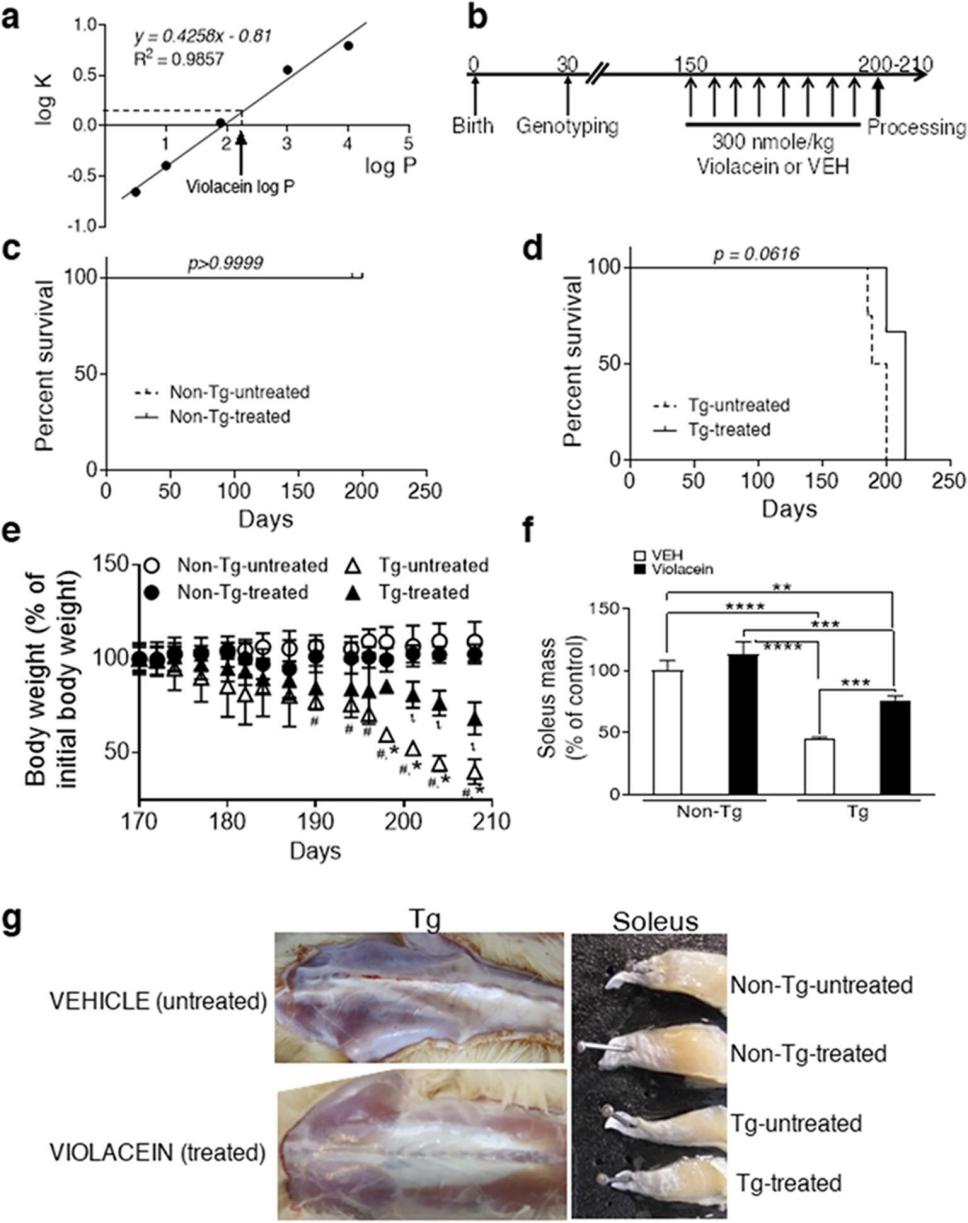
Violacein: log P determination and in vivo treatment. (**a**) Linear correlation between standard´s log K versus log P and interpolation of violacein log P evidencing its potential capacity to cross the blood brain barrier. (**b**) Scheme of animal treatment performed in 150 day-old Non-Tg and Tg rats that received intraperitoneal injections of 300 nmole/kg violacein (300 µl) (called as –treated animals) or equal volume of vehicle (0.5% DMSO, called as -untreated rats). (**c**) Survival Kaplan-Meir curves showing absence of violacein effects in the lifespan of Non-Tg rats (p > 0.9999, Long-rank Mantel-Cox test). (**d**) Survival Kaplan-Meir curves of Tg rats showing a marginally significant delayed lifespan upon violacein treatment (~ 10 days, p = 0.0616, Long-rank Mantel–Cox test). (**e**) Body weight curve between 170 and 210 day-old for all experimental conditions. Differences related to Non-Tg rats that determined disease onset^16^ became statistically significant at 180 day-old (#, p = 0.0374) for Tg-untreated (△), and at 190 day-old (ι, p = 0.030) for Tg-treated (▲) rats, respectively. Different body weight loss between Tg-treated (▲) and Tg-untreated (△) animals was found at 194 day-old (*, p = 0.041). Violacein did not affect the body weight of Non-Tg treated (●) versus Tg-untreated rats (○). (**f**) Decreased soleus muscle mass in Tg-untreated related to Non-Tg rats (~ 50%, p < 0.0001), and attenuation of muscle loss in Tg-treated when compared with Non-Tg rats (~ 20%, p = 0.0001). (**g**) Significant reduced atrophy in gluteus muscles from a Tg-treated rat when compared with an age-matched Tg-untreated brother. On the right there are halves of soleus muscles from each experimental condition. Note the preservation of muscle mass when comparing Tg-treated with Tg-untreated samples. Numbers of animals employed and replicates done appear in Table 1.

Based on previous evidence reporting that violacein could cross LPS-injured blood brain barrier (BBB)^30^, here we predicted its ability to cross the intact BBB^38,39^. Results obtained showed that violacein has no ionization in the physiological pH range and that its log P value (2.24, calculated by RP-HPLC) is within the optimal range for BBB penetration (Fig. 2a; Table 2)^38,39^. In addition, the molecular weight (343.3), the number of hydrogen bond donors (4) together with the sum of all nitrogens and oxygens (6) indicates that violacein´s molecular structure fulfills the thresholds of the Lipinski’s rules for molecules able to cross the BBB^39^.

### Violacein effects on lifespan and disease onset

The dose of violacein (300 nmole/Kg/week), chosen because of the AbAs selective vulnerability over AA (Fig. 1c–h), was applied (Fig. 2b) weekly due to less animal handling and similar results than divided doses. Animals that received vehicle were called as –untreated whereas those that received violacein were named as –treated.

The treatment applied did not cause visible effects in Non-Tg animals including unchanged lifespan (p > 0.9999) (Fig. 2c) or movement (mark 5/5 in the disease progression scale^16^). Regarding Tg rats, violacein effects on survival were marginally significant when comparing –untreated with –treated groups (p = 0.0616), and the survival median determined by Log-rank Mantel-Cox test passed from 198.5 to 215.0 day-old (Fig. 2d). Violacein did not influence the body weight of Non-Tg animals (Fig. 2e). Instead, it marginally delayed the onset of body weight loss^5^ (p = 0.0900) in Tg-treated (~ 190 day-old, ι, p = 0.0300) versus –untreated animals (~ 180 day-old, #, p = 0.0374) (Fig. 2e). In addition, body weight differences in Tg-treated versus Tg-untreated rats reached statistical signification at 194 day-old (*, p = 0.0410) and lasted up to the end stage (Fig. 2e).

First abnormal gait (mark 4/5) was detected at ~ 174–180 day-old for Tg-untreated and Tg-treated rats, whereas paralysis of a hind limb (mark 3/5) was seen at 178 ± 5 day-old in Tg-untreated rats and was marginally statistically delayed until 185 ± 5 day-old (p = 0.0900) in Tg-treated rats. A marginally significant delay was also observed when both limbs became paralyzed (mark 2/5) in Tg-untreated (187 ± 5 day-old) versus Tg-treated rats (200 ± 7 day-old) (p = 0.0770), respectively. Dates of end stage (mark 1/5), recognized as the inability to recover right position when turned on their back, also was marginally significant delayed and occurred at 195 ± 9 and 210 ± 10 day-old for Tg-untreated versus –treated rats (p = 0.0900), respectively.

Once determined the effects of the protocol applied on the survival and disease progression of Tg animals, we decided to analyze the effects of the dose applied on some of the main ALS signs (muscle atrophy, NMJ integrity and spinal cord decreased motor neuron density and increased glial reactivity) when Tg-untreated animals reached the end stage. At that time, animals from each experimental condition were simultaneously processed and samples obtained to perform the planned approaches.

Masses of soleus muscles dissected at the same time for each experimental condition indicated lack of violacein effects in Non-Tg rats (640 ± 113 mg and 600 ± 100 mg) for -untreated versus treated animals, p = 0.3395. Instead, Tg-treated rats showed soleus muscles heavier than Tg-untreated brothers (420 ± 30 mg versus 320 ± 7 mg, p < 0.001) (Fig. 2f). Thus, soleus mass of Tg rats was preserved in ~ 20% upon violacein treatment. In accordance, besides maintaining muscle mass in general, violacein significantly improved the gross morphology of lower limb muscles in Tg-treated rats (Fig. 2g, bottom photograph ) when comparing with age-matched Tg-untreated animals (Fig. 2g, upper photograph). It also caused that soleus muscles from Tg-treated rats had an appearance closer to those of Non-Tg animals (Fig. 2g).

### Violacein partially protected muscle fibers and NMJ integrity in Tg rats

**Figure 3:**
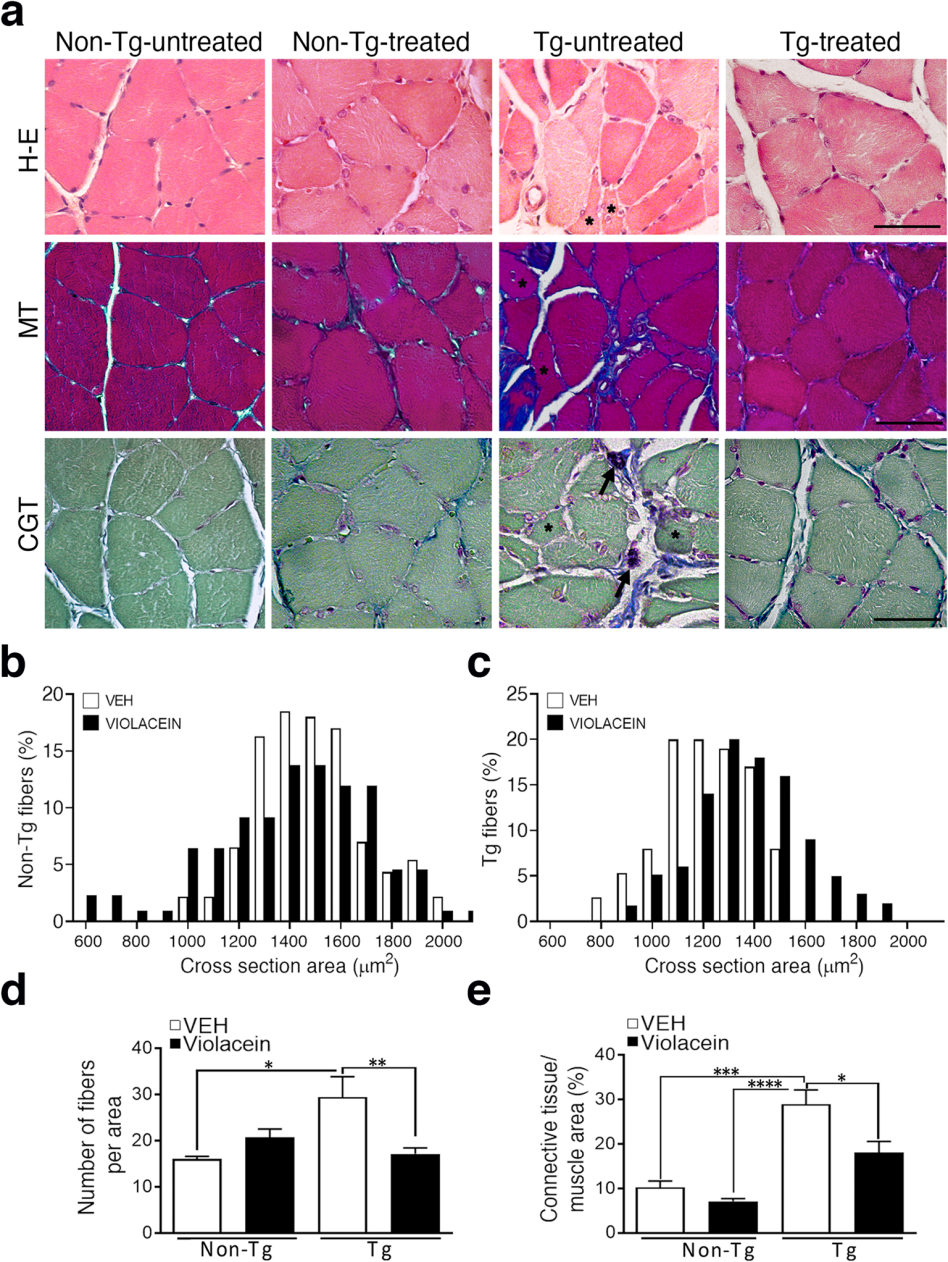
Histological analysis of muscle soleus upon violacein treatment. (**a**) Representative light images of cross-sectional muscle fibers stained with hematoxylin–eosin (H&E) and Masson (MT) and Cajal-Gallego (CGT) trichromes from each experimental group. Non-Tg animals showed muscle fibers of similar size and morphology and ordered collagen (blue staining in MT or CGT). Instead, Tg-untreated condition presented atrophic fibers (asterisks), fibrosis evidenced by abundant disordered collagen fibers and mastocytes (black arrows in CGT staining) close to atrophic fibers indicating clear pathological signs. The Tg-treated condition showed minor pathological signs as indicated by less atrophic fibers, decreased collagen areas and general appearance closer to that of Non-Tg conditions. Calibration: 50 μm. (**b**, **c**) Distribution of soleus cross sectional areas in Non-Tg (**b**) and Tg (**c**) -untreated (vehicle, white columns) and -treated (violacein, black columns) rats showing in Tg-treated animals a displacement of the fiber size frequency peak toward the bigger values. (**d**) The number of fibers per area were increased in Tg-untreated related to Non-Tg (p = 0.0338) or Tg-treated rats (p = 0.0099). Non-Tg and Tg-treated values were similar (p = 0.2229). (**e**) Quantitative analysis of MT stain evidenced increased collagen in Tg-untreated when compared with Non-Tg (p = 0.0007) or with Tg-treated rats (p = 0.0175).

To determine whether violacein helps to prevent the ALS abnormal muscular features that are recapitulated in hSOD1G93A models^5,51–57^; H&E and trichrome stainings were made in soleus muscles from each experimental condition (Fig. 3). In comparison with Non-Tg conditions, the Tg-untreated muscle section evidenced clear pathological signs that include the presence of atrophic fibers and degranulating mastocytes (asterisks), reduced muscle area and increased collagen deposition (blue in mid and bottom images). Instead, the muscle section from Tg-treated animals exhibited better uniformity in fiber size and shape (Fig. 3a, upper images), less connective tissue (Fig. 3, mid and bottom images), and a general appearance closer to that of Non-Tg animals. Morphometric analysis showed similar distribution of cross sectional areas in Non-Tg rats, independent on violacein treatment (Fig. 3b left). However, in Tg rats, there was a shift in the frequency toward bigger cross sections when comparing –treated with –untreated animals (Fig. 3b right). Violacein also abrogated the ~ 30% increased number of muscle fibers per area in Tg-treated versus –untreated rats (p = 0.0099) (Fig. 3c), carrying the Tg-treated values similar to those of Non-Tg animals (p = 0.338). Regarding collagen, Tg-untreated and Tg-treated showed ~ 190% (p = 0.0007) and ~ 100% (p = 0.0175) increased collagen deposition than Non-Tg rats (Fig. 3d).

**Figure 4:**
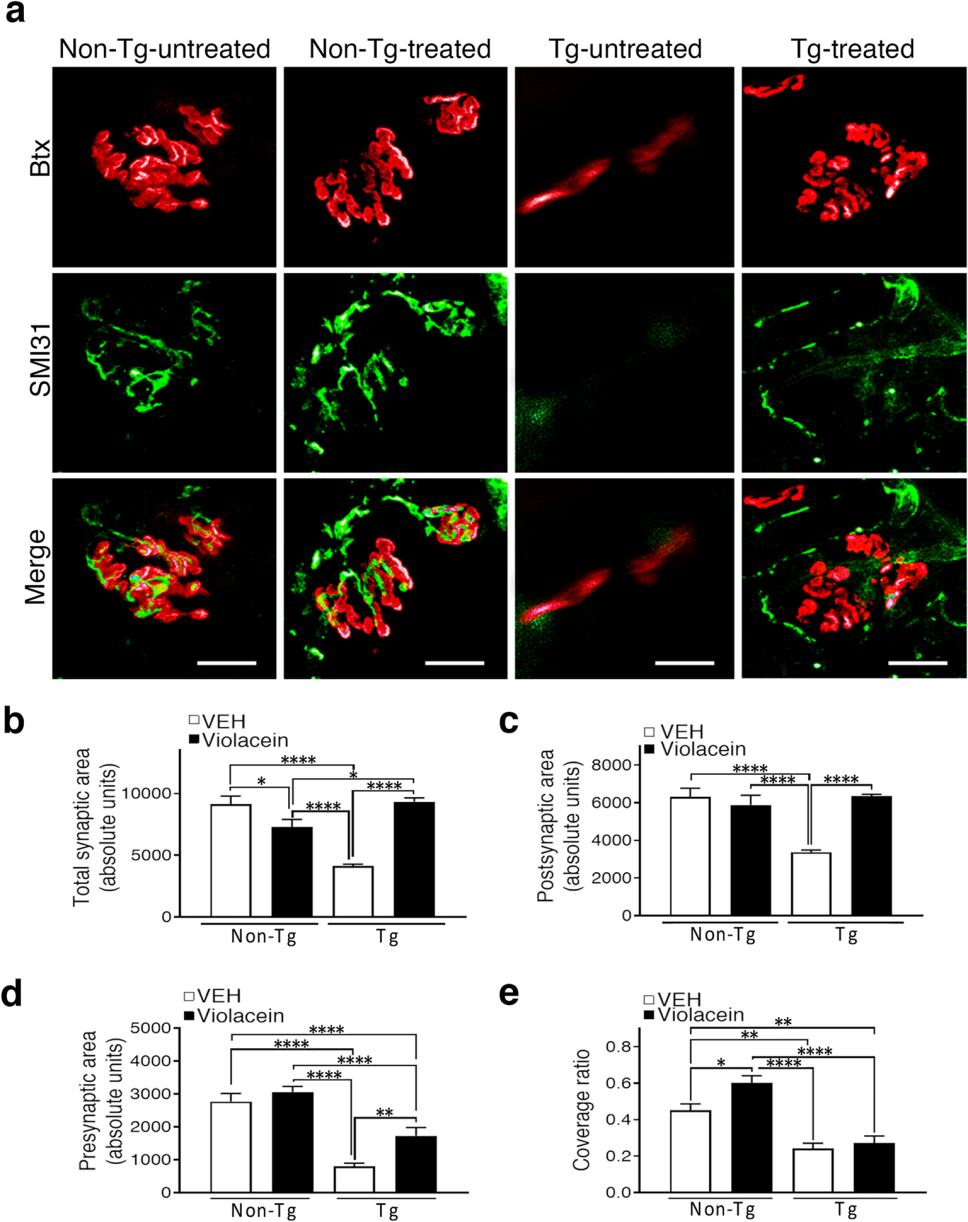
Violacein effects on soleus NMJs. (**a**) Representative confocal Z-stack images of NMJ immunofluorescences from each experimental condition. The alignment between the overlying nerve terminals labelled with SMI31 (green) and underlying acetylcholine receptors labelled with αBungarotoxin (Btx, red) is clearly shown in Non-Tg rats. The almost complete disappearance of both NMJ components is observed in Tg-untreated rats. Instead, Tg-treated showed a notable preservation of the postsynaptic element (pretzel shape) together with an improved signal of the presynaptic component. Calibration: 20 µm. (**b**) Quantitation of the total synaptic area showed decreases in Tg-untreated versus Non-Tg (p < 0.0001) or Tg-treated rats (p < 0.0001) and no differences between Tg-treated and Non-Tg rats (p > 0.9999). (**c**) Quantitation of Btx positive postsynaptic areas showed decreases  in Tg-untreated related either to Non-Tg or to Tg-treated (p < 0.0001) rats, and similar values in Non-Tg versus Tg-treated samples (p > 0.9999). (**d**) Assessment of the phosphorylated NMJ presynaptic component indicated decreased values in Tg-untreated as well as in Tg-treated versus Non-Tg rats (p < 0.0001 for both comparisons). However, Tg-treated showed the half of the loss seen in Tg-untreated animals. Thus, violacein allowed that Tg-treated rats almost preserved a duplicated innervation area when compared with Tg-untreated animals (p = 0.0084). (**e**) Quantitative analysis of the coverage ratio indicated that violacein did not influence this parameter in Tg-treated versus Tg-untreated rats (p = 0.9400).

When analyzing violacein actions on the NMJ architecture and components (Fig. 4), there were no effects on Non-Tg animals. However, positive impact on Tg-treated animals included a significant preservation of the typical architecture (pretzel shape)^47–49,52^ of the postsynaptic component labelled with αBungarotoxin (Btx) that had an appearance similar to that of Non-Tg rats. When comparing Tg-treated with–untreated conditions, violacein partially improved the presynaptic NMJ component as indicated by the higher SMI31 signal that labels nerve terminals (Fig. 4a). Upon violacein treatment, quantitation corroborated the preservation of Btx positive areas (p < 0.0001) and total synaptic areas (p < 0.0001) (Figs. 4b, c), as well as the enlarged SMI31 areas in Tg-treated versus Tg-untreated rats (p = 0.0084) (Fig. 4d). However, the coverage ratio was not modified in Tg-treated versus –untreated rats (p = 0.9400), and remained as ~ 50% of coverage shown in Non-Tg rats (Fig. 4e).

**Figure 5:**
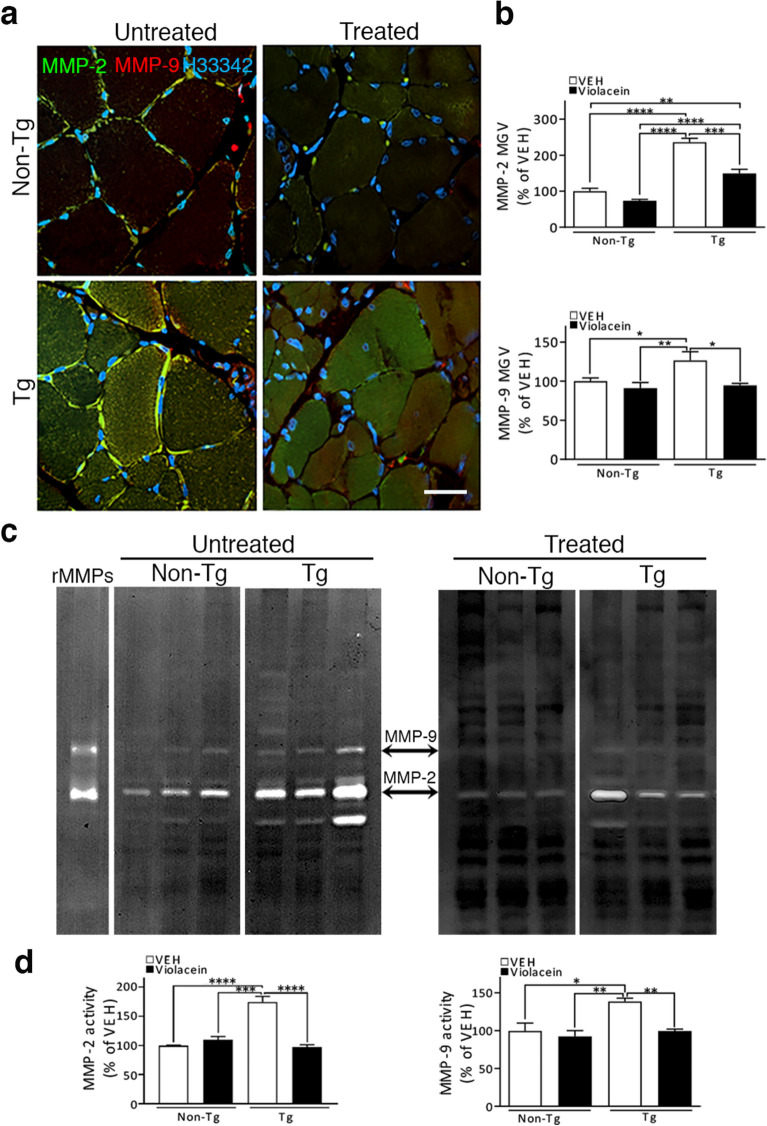
Violacein inhibited muscle MMP-2 and -9. (**a**) Representative confocal immunofluorescences of soleus muscle transverse sections labelled against MMP-2 (green) and MMP-9 (red). Non-Tg muscles showed positive signals associated to the sarcolemma. The same location but increased signal appeared in the Tg-untreated muscle together with a punctate expression in the sarcoplasm. Instead, Tg-treated sections showed low signals associated to the sarcolemma plus a heterogeneous staining in the sarcoplasm with more atrophic fibers enriched in MMP-9 (red). Cell nuclei were labelled with Hoescht 33342. Calibration: 20 µm. (**b**) Quantitation of violacein inhibitory effects on MMP-2 and -9 immunoreactivity assessed by MGV. Upper chart indicates that MMP-2 increased in Tg-untreated (~ 130%, p < 0.0001) and –treated (~ 80%, p < 0.0001) versus Non-Tg samples. MMP-9 (bottom chart) was increased in Tg-untreated versus Non-Tg samples (~ 50%, p = 0.0455), but had similar values in Tg-treated and Non-Tg samples (p = 0.09). (**c**) Gel zymograms showing MMP-2 and MMP-9 gelatinolytic activities in soleus homogenates from 3 Non-Tg and 3 Tg animals that received vehicle (-untreated, left zymogram) or violacein (-treated, right zymogram). Note that violacein decreased both gelatinase signals. On the left appears an isolated lane showing the zymogram of the controls (recombinant MMP-2 and -9) that were ran in each gel. (**d**) Quantitation of violacein inhibitory effects of MMP -2 and -9 activities. Left chart shows increased MMP-2 activity when comparing Tg-untreated with either Non-Tg (p < 0.0001) or Tg-treated (p = 0.0003) samples. Tg-treated and Non-Tg homogenates showed similar MMP-2 activity (p = 0.7025). Right chart shows the increased MMP-9 activity in Tg-untreated versus Non-Tg (p = 0.0128) or versus Tg-treated (p = 0.0072) samples. Non-Tg and Tg-treated samples showed similar values (p = 0.0900).

As MMP-2 and -9 play significant roles in neuroinflammation and were increased in ALS in close relation with progressive atrophy^32,33,48^, we have tested violacein effects on MMP-2 and -9 immunoreactivities and activities in soleus muscle samples (Fig. 5). Immunofluorescence (Fig. 5a) showed positive signals associated to sarcolemma in Non-Tg muscle sections but Tg rats also showed punctate immunoreactivity inside the muscle fibers. Violacein caused a general decrease in MMP-2 intracellular immunoreactivity in Tg –treated versus –untreated animals (p = 0.0012) (Fig. 5b). Similar findings were found in MMP-9 signals, with violacein abrogating the increase seen in Tg-untreated samples and leading their values closer to those of Non-Tg animals (p = 0.0455) (Fig. 5b).

Zymography assays (Fig. 5c, d) showed increased MMP-2 activity in muscle homogenates from Tg-untreated versus  Non-Tg-untreated (left zymogram) or versus Tg-treated (right zymogram) (p < 0.0001) samples. Violacein effects were better seen when quantitating MMP-2 activity in Tg-treated compared with Tg-untreated samples (~ 50% decrease, p < 0.0001), and with similar activity values between Tg-treated and Non-Tg samples (p = 0.7025). Regarding MMP-9 activity, although it was much lower than that of MMP-2, violacein decreased it as seen when comparing samples from Tg–treated with –untreated rats (p = 0.0072).

### Violacein controlled inflammation and glial reactivity in the spinal cord of Tg-treated rats

**Figure 6:**
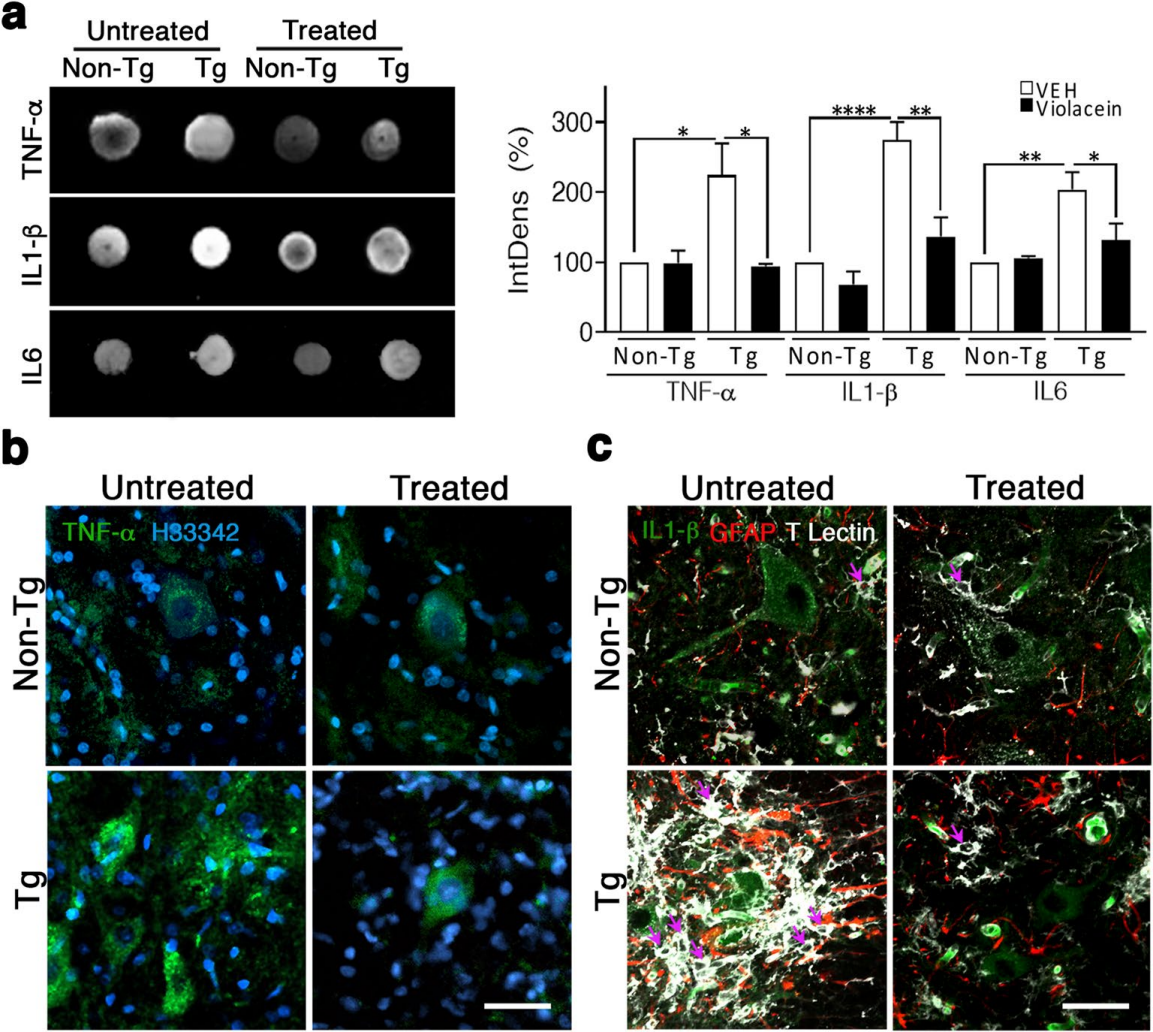
Low levels of inflammatory cytokines in spinal cord of Tg-treated rats. (**a**) Dot blot assays showed TNF-α, IL-1β and IL6 increased signals in spinal cord homogenates of Tg-untreated versus Non-Tg rats, and decreased signals upon violacein treatment. The chart showing the quantitated integrated density parameterized to Non-Tg-untreated samples confirmed increased values in Tg-untreated for TNF-α (p = 0.0197), IL-1β (p < 0.0001), and IL6 (p = 0.0015), respectively. Similar levels were seen in Tg-treated versus Non-Tg samples for TNF-α (p = 0.9991), IL-1β (p = 0.1905) and IL6 (p = 0.7290), respectively. The amount of protein used per spot was 30 µg. (**b**) Representative confocal Z-stack images of TNF-α immunofluorescences in spinal cord sections from each experimental condition. Low but specific cytoplasmic signal (green) was seen in some motor neurons from Non-Tg rats independently on violacein treatment. Instead, in Tg-untreated section, TNF-α brighter signals were observed in more motor neurons and widespread inside the spinal cord parenchyma. In the Tg-treated section, TNF-α staining was very similar to that of Non-Tg samples. Cell nuclei were labelled with Hoechst 33342. (**c**) Z-stack images of immunofluorescences against IL-1β (green), GFAP (red) and Tomato lectin (white) from all experimental groups. Non-Tg sections showed low but specific IL-1β expression in motor neurons and blood vessels. No significant signals were seen in astrocytes or microglial cells (magenta arrows). Instead, in the Tg-untreated section, more IL-1β positive motor neurons were seen as well as extracellular positive signals together with exacerbated glial reactivity throughout the spinal cord parenchyma. Some reactive astrocytes may express low IL-1β levels as suggested by the presence of yellow/orange spots. Exacerbated reactive microglia appeared with swollen cell bodies and negative to IL-1β. Upon violacein treatment, IL-1β signal in the Tg-treated section seemed restricted to motor neurons at lower levels than Tg-untreated samples and blood vessels remained highly positive. In this section, glial reactivity was dramatically lower than in Tg-untreated samples as evidenced by the presence of few hypertrophic astrocytes and microglial cells with signals of low/mid reactivity. Calibration: 50 µm (**b**) and 40 µm (**c**), respectively.

Dot blots of Tg-untreated spinal cord homogenates showed increased levels of TNF-α (~ 124%, p = 0.0197), IL-1β (~ 175%, p < 0.0001) and IL6 (~ 103%, p = 0.0015) when compared with Non-Tg samples (Fig. 6a). Instead, no differences were found between Tg-treated and Non-Tg samples for TNF-α, IL-1β and IL6 as indicated by the respective p values (p = 0.9991; p = 0.1905 and p = 0.7290) (Fig. 6a chart). In addition, when comparing Tg-untreated with -treated rats, violacein abrogated the increases in TNF-α (p = 0.0406), IL-1β (p = 0.0015) and IL6 (p = 0.0373) levels (Fig. 6a). Consistently, TNF-α immunoreactivity in the spinal cord sections from Tg-treated rats was low and restricted to the motor neuron cytoplasm, whereas Tg-untreated rats showed more intense signals in motor neurons and in the extracellular parenchyma (Fig. 6b). No evident changes attributed to violacein were found in the TNF-α immunoreactivity of Non-Tg spinal cord sections.

Regarding IL-1β, immunofluorescences showed positive signals inside the motor neurons and in blood vessels, but not in astrocytes or microglia of Non-Tg and Tg-treated spinal cords (Fig. 6c). Instead, in Tg-untreated rats, IL-1β was increased in motor neurons and seemed detected at very low levels in reactive astrocytes but not in reactive microglia (Fig. 6c, red and white cells). IL-1β immunoreactivity in Tg-treated spinal cord exhibited similar levels and localization than Non-Tg sections and was accompanied by significant lower glial reactivity as evidenced by GFAP and Tomato lectin staining (Fig. 6c).

**Figure 7:**
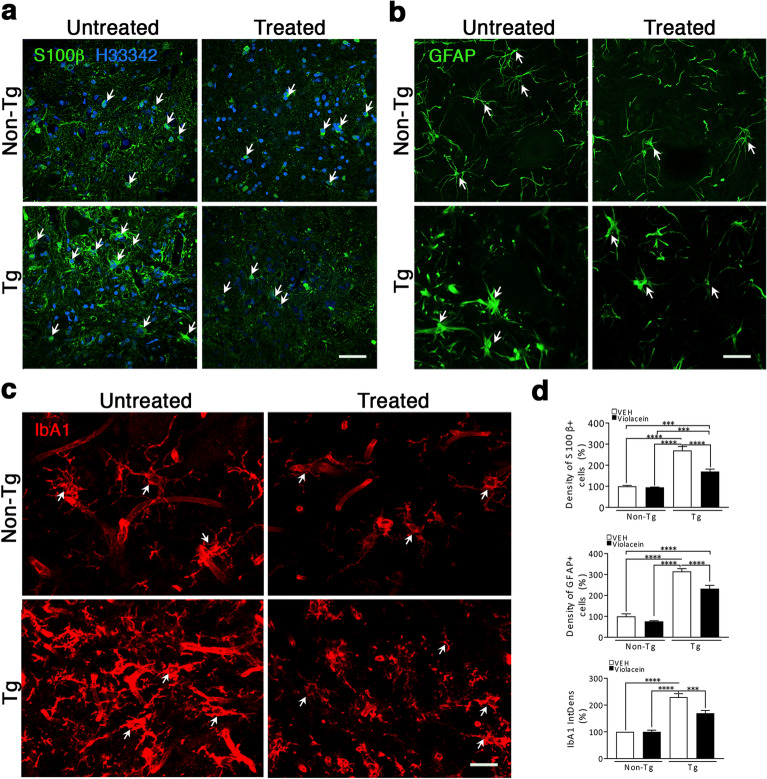
Very low glial reactivity in the spinal cord of Tg-treated animals. (**a**) Representative confocal Z-stack images of S100β immunofluorescence (green) in each experimental condition. Whereas in Non-Tg images, S100β appeared surrounding the nucleus, there was an increased density of positive swollen cells with coarse processes in Tg-untreated rats. Violacein treatment modulated both the number and appearance of S100β positive cells in the Tg-treated condition. Hoechst 33342 stained cell nuclei (blue). (**b**) Representative Z-stack images of GFAP immunofluroescences in all experimental conditions. Non-Tg and Tg-treated images exhibited delicate positive cell processes whereas in Tg-untreated rats there was a predominance of GFAP positive swollen cells with gross and short positive processes. (**c**) Confocal Z-stack images of the microglial cell marker Iba1 in all experimental conditions. Images from Non-Tg and Tg-treated sections showed positive cells with thin processes whereas in the Tg-untreated image there was an increased signal mostly present in swollen cells with short and coarse processes. Blood vessels were delicately positive to Iba1 . For each marker analyzed ((**a**), (**b**) and (**c**)), white arrows point positive cells representative of each experimental condition. Calibration: 100 µm, 40 µm and 20 µm for (**a**), (**b**) and (**c**), respectively. (**d**) Quantitation of S100β, GFAP and Iba1 cellular density related to Hoechst 33342 positive cells indicated that Tg-untreated versus Non-Tg samples had increases in S100β (p < 0.0001), GFAP (p < 0.0001) and Iba1 (p < 0.0001), respectively. Violacein caused less but yet significant increased values in Tg-treated versus Non-Tg conditions as indicated by S100β (p < 0.0001), GFAP (p < 0.0001) and Iba1 (p < 0.0001), respectively. In all cases, quantitation was done by measuring the cells that co-localized with Hoechst 33342 positive cell nuclei. Hoechst 33342 signal was omitted to emphasize violacein modulation of GFAP and Iba1 immunoreactivity in (**b**) and (**c**), respectively.

Exploration of glial reactivity upon violacein treatment in lumbar spinal cord sections (Fig. 7) evidenced that Non-Tg (independent on violacein treatment) and Tg-treated animals exhibited astrocytes and microglial cells without any sign of reactivity. Instead, prominent astrocytosis (Fig. 7a, b) and microgliosis (Fig. 7c) appeared in Tg-untreated animals as evidenced by abundant swollen cells with short coarse processes (arrows) as well as by increased density of S100β (~ 170%, p < 0.0001), GFAP (~ 150%, p < 0.0001) and Iba1 (~ 190%, p < 0.0001) positive cells when compared with Non-Tg values (Fig. 7d). Tg-treated condition not only showed astrocytes and microglial cells very similar to those from Non-Tg conditions (Fig. 7a-c) but also minor increases in cellular density of S100β (~ 70%, p < 0.0001), GFAP (~ 50%, p < 0.0001) and Iba1 (~ 70%, p < 0.0001), respectively.

**Figure 8:**
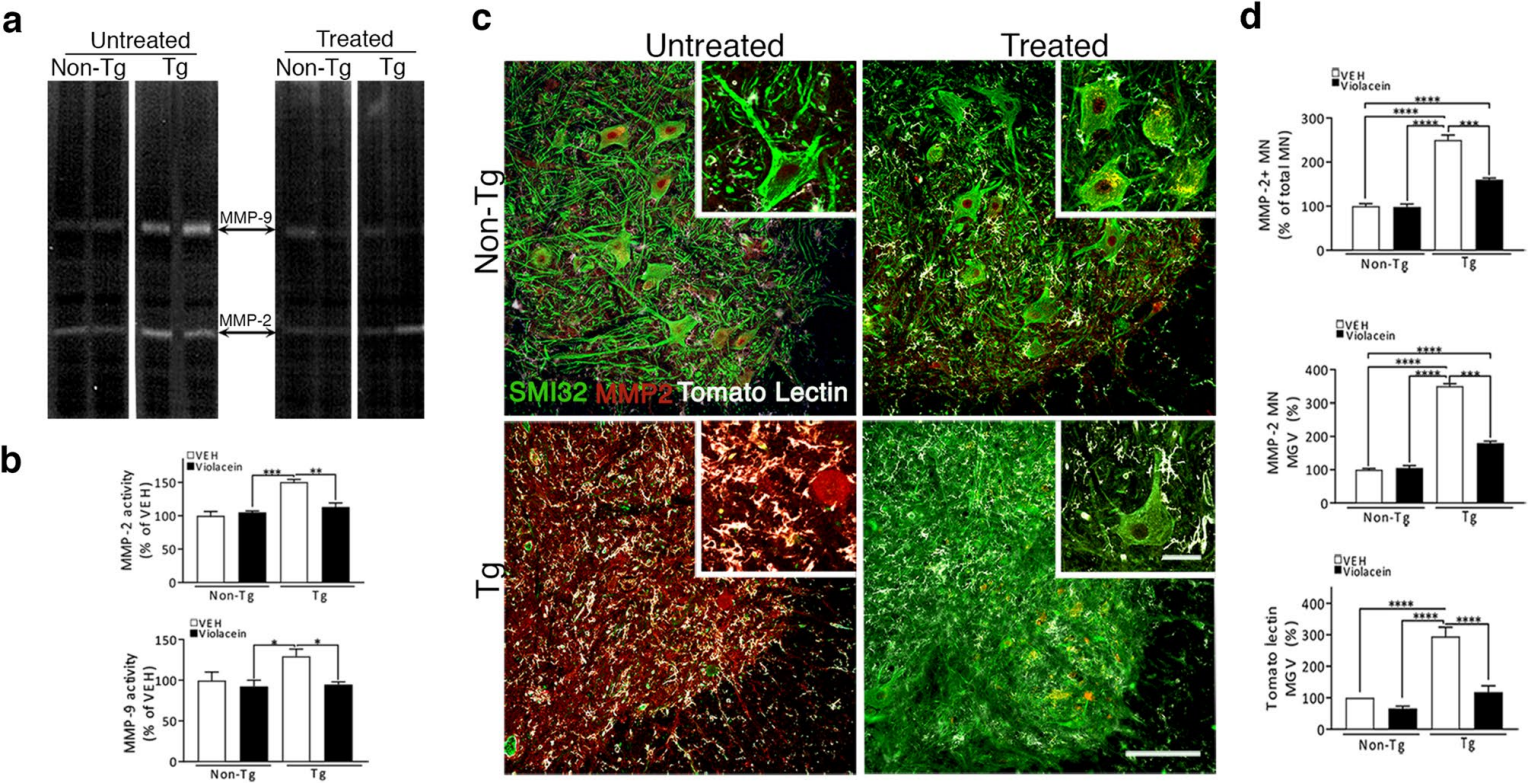
Low MMP-2 activity and immunoreactivity in lumbar spinal cord from Tg-treated rats. (**a**) Zymograms from spinal cord homogenates from all experimental conditions evidenced violacein effects decreasing MMP-2 and -9 signals. (**b**) Quantitative analysis showed increased MMP-2 (p = 0.0005) and MMP-9 (p = 0.0292) activities in Tg-untreated compared with Non-Tg homogenates, but also in Tg-untreated versus Tg-treated samples (p = 0.0016 and p = 0.0115, for MMP-2 and MMP-9, respectively). No differences were found in MMP-2 and MMP-9 activity between Tg-treated and Non-Tg samples (p = 0.7945 and p = 0.9987, respectively). (**c**) Representative confocal Z-stack images of immunofluorescences against MMP-2 (red), SMI32 (green) and Tomato lectin (white) on transverse sections of lumbar spinal cords from each experimental condition. In Non-Tg sections appeared many SMI32 positive motor neurons with typical morphology and with nuclear and perinuclear MMP-2 signal. Higher MMP-2 immunoreactivity was seen in Tg-untreated section mostly in motor neurons with low SMI32 expression and also in the spinal cord parenchyma. This was accompanied with high microglial reactivity and generally decreased SMI32 immunoreactivity. The Tg-treated condition exhibited less MMP-2 positive motor neurons, more SMI32 positive motor neurons with the typical signal^45^ as well as lower microglial reactivity when compared with Tg-untreated condition. Insets in each image show motor neurons with representative SMI32 and MMP-2 signals found in each condition and microglia with different degrees of reactivity. Calibration: 30 µm (images) and 15 µm (insets). (**d**) Quantitation of the immunoreactivity signals indicated a decreased percentage of MMP-2 positive motor neurons related to total SMI32 positive motor neurons as well as significant decreased MMP-2 positive motor neurons when comparing Tg-treated versus Tg-untreated rats (p = 0.0001). A remarkable decrease in microglial reactivity assessed by MGV of Tomato lectin was determined in Tg rats upon violacein treatment (p < 0.0001).

In addition, analysis of MMP-2 and -9 in spinal cord indicated that Tg-untreated homogenates showed increased MMP-2 (~ 50%, p = 0.0005) and MMP-9 (~ 40%, p = 0.0292) gelatinase activities related to Non-Tg or to Tg-treated samples (p = 0.0016 and p = 0.0115 for MMP-2 and MMP-9, respectively). Instead, no differences were found between Non-Tg and Tg-treated samples either for MMP-2 (p = 0.4719) or MMP-9 (p = 0.9960), respectively (Fig. 8a, b). MMP-2 expression was clearly observed in SMI32/MMP-2/Tomato lectin immunofluorescences of spinal cord sections from each experimental condition, but clearly increased in that from Tg-untreated rats (Fig. 8c).

The MMP-2 signal (red) inside and around the nucleus of motor neurons highly positive to SMI32 (green) was similar in Non-Tg and Tg-treated conditions. Instead, Tg-untreated sections showed extremely increased MMP-2 in motor neurons, mainly in those that lack SMI32 signal^49^, and in the rest of the spinal cord parenchyma including the highly reactive microglial cells that were recognized for the swollen bodies positive to Tomato lectin (white, Fig. 8c). Tg-treated sections also appeared with more SMI32 positive motor neurons than Tg-untreated condition. Quantitation of MMP-2 levels in Tg-treated versus Tg-untreated sections showed a minor percentage of positive motor neurons (from ~ 100% decreased, p = 0.0009) and lower signals (~ 250% decreased, p = 0.0001) (Fig. 8d). In addition, microglial  reactivity was reduced in Tg-treated when compared with Tg-untreated rats (~ 200% decrease, p < 0.0001) (Fig. 8d).

**Figure 9:**
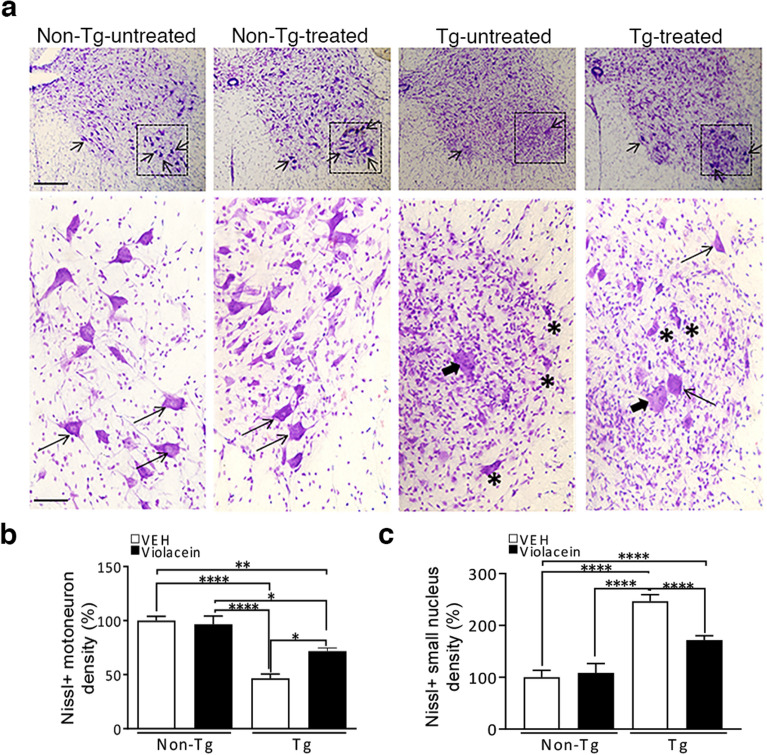
Histological evidence of the modulation of motor neurons and glial cells upon violacein treatment. Nissl staining of spinal cord transverse sections from each experimental condition allowed analyzing the morphology and density of motor neurons in the IX Rexed lamina. (**a**) Upper images show panoramic views of spinal cord hemisections with motor neurons that are distinguishable by size and intensity of the signal (black arrows). Bottom images show higher magnifications of the motor neurons present in the dashed area of each condition. Typical appearance were found in Non-Tg samples in contrast to the isolated swollen motor neuron found in Tg-untreated samples (thick short arrow) together with degenerating motor neurons (asterisks) and a plethora of small intensely stained nuclei likely corresponding to glial cells. Tg-treated sections showed a bigger density of motor neurons and decreased glial cell density as evidenced by less amount of small nuclei stained. Calibration: 200 µm and 20 µm for upper and bottom images, respectively. (**b**, **c**) Quantitation of the density of motor neurons (**b**) and glial cell nuclei (**c**) in the IX Rexed lamina confirmed that violacein treatment preserved motor neurons and impaired increased density of glial cells. Statistically significant differences were found among Tg–treated versus –untreated conditions for motor neuron and glial cell densities (p < 0.0001).

Nissl staining of motor neurons in IX Rexed lamina showed that violacein treatment preserved the morphology of motor neurons (Fig. 9a) and that seemed responsible for the increased motor neuron density in Tg-treated versus Tg-untreated rats (~ 50%, p = 0.0111), although the last one did not reach the values shown in Non-Tg animals (~ 25% less, p = 0.0024) (Fig. 9b). Remarkably, compared with Tg-untreated samples, Tg-treated showed much less small intensely labeled nuclei corresponding to glial cells surrounding motor neurons. Determination of the number of these small nuclei  confirmed a decreased glial cell density when comparing Tg-treated with Tg-untreated samples (~ 70% less, p < 0.0001) (Fig. 9c). Thus, the different approaches employed allow suggesting that violacein not only controlled neuroinflammation, it partially preserved motor neurons, and also impaired glial cell reactivity, all enlarging its protective repertoire displayed in the ALS model employed.

## Discussion

Natural products are attractive sources of therapeutic agents, with the majority of commercially available drugs derived from microorganisms, plants and animals because of their multiple beneficial actions^18–22,30^. Violacein is a quorum sensing metabolite extracted from different bacterial strains, including the *Janthinobacterium sp.* UV13^17^, that bears anti-inflammatory, anti-proliferative, anti-tumor and pro-apoptotic properties in experimental models of many diseases^17,18,21,23–25^. Here, we have tested if violacein could be a potential therapeutic candidate to control aberrant invading glial phenotypes that have deleterious roles in neurodegenerative diseases. Next, we studied whether violacein could exhibit protective effects on the rat hSOD1G93A ALS model.

Evidence obtained shows that violacein controlled AbAs, the aberrant glial cells that emerge and proliferate in the degenerating spinal cord of the hSOD1G93A (Tg) rats and do not suffer replicative senescence once in culture^11,13^. Present data reinforces the existing literature ^14,16,51^ about the vulnerability of these aberrant glial phenotypes^11,13^ to anti-proliferative, anti-inflammatory or antioxidant compounds^18,22–30^. However, violacein reduced AbAs viability at concentrations significantly lower than those employed in  many cell lines including malignant glioma cells^21–28^. Remarkably, at the concentrations that affected AbAs, there were no significant effects on the viability and functional parameters of astrocytes obtained from Non-Tg adult rats. This evidencing a therapeutic concentration window that may allow to control the aberrant glial phenotypes without disturbing the cells responsible for the maintenance of CNS homeostasis^34^.

Moreover, violacein concentrations used to kill AbAs were much lower than those of the anti-proliferative drugs previously employed in vitro and in mouse and rat hSOD1G93A models^16,51^, suggesting an impressive capacity to control AbAs through many potential mechanisms. It has been reported that violacein induced apoptosis in some cancer cells through oxidative stress and imbalanced antioxidant defenses^19,23,25^. Our results showed that AbAs increased oxidative stress and reduced glutathione levels at 200 nM, a concentration close to the IC_50_ (~ 175 nM) determined in viability assays.  Thus, oxidative stress may be a potential mechanism underlying the violacein effects on AbAs survival. We have also found alterations in mitochondrial functionality and potential at 30 and 50 nM violacein, suggesting it aggravated the AbAs basal mitochondrial dysfunction altering even more their capacity to obtain energy efficiently^34^. It is also possible that violacein may increase the exacerbated ER stress seen in AbAs^14^ or even impaired their alternative energetic sources^34^, that are already stressed by mitochondrial dysfunction. All underscoring the violacein potential capacity to kill AbAs via different underlying mechanisms that need to be precisely identified.

The evidence that indicates that violacein could cross the intact BBB^37–39^ was other remarkable finding and represents a valuable advantage from the therapeutic perspective. In this regard,  when assayed in animals, violacein caused marginally significant delays in the lifespan and in disease progression of Tg-treated rats. The p values resulting from the statistical analysis of both parameters  suggest that modifications in the experimental paradigm employed, either by starting it earlier or by increasing the periodicity of administrations, could allow to reach a longer lifespan or slow disease progression. Although the possibility of increasing the dose of treatments needs to be analyzed in depth if desired to selectively preserve homeostatic astrocytes and likely the other neural cells, the dose employed in this work (300 nmole/Kg, ~ 0.1 mg/Kg) is much lower than those reported as toxic  (7–10 mg/Kg)^25,30^. Then, doses might be carefully increased if the greater periodicity of administrations or the treatments in younger animals fail.

Weekly administrations of 300 nmole/Kg to Tg animals resulted in better preserved muscles and NMJs as well as more spinal motor neurons, suggesting that violacein has a potential capacity to slow disease progression^5^. Related to Tg-untreated animals, Tg-treated rats showed minor loss of muscle mass as reflected in soleus bigger fiber cross sections and decreased collagen areas as well as significantly preserved NMJ components. In ALS has been described an augmented production of extracellular matrix components, especially collagens, that will lead to cumulative fibrosis^52^, a pathological process that very recently has been suggested as a common trait that correlates with disease progression^53^. It also has been reported that fibrosis in skeletal muscles impairs function and regeneration  and is a main cause of muscle weakness^54^. Therefore, the control of  collagen in Tg-treated rats seems other violacein relevant effect.

Regarding NMJ, its dismantling is reported as an early event in ALS^52,55–57^. Recently, it also has been proposed that there is a long time window after the onset of NMJ loss in which motor neurons are not globally degenerating and preserve their capacity to re-innervate NMJs^56,57^. Apart from the protective effects on the NMJ components, upon violacein treatment, the coverage ratio was similar in Tg-treated and Tg-untreated animals, suggesting that the synaptic function was not preserved. Thus, a new experimental paradigm also needs to extend the positive effects to the NMJ in order to preserve not only its architecture but also its functionality.

The neuroprotective effects upon violacein treatment were also observed at level of the spinal cord of Tg rats, particularly by the decreased levels of the inflammatory cytokines that were predominantly expressed in motor neurons as well as by the inhibition of two glial reactivity hallmarks, the typical morphological changes and the increased cell proliferation. A higher density of motor neurons at level of IX Rexed laminae together with decreased number of MMP-2 positive motor neurons in Tg-treated rats were also observed. These results seemed in accordance with violacein neuro-immunomodulatory and anti-inflammatory properties^22,30^, including the reduction of systemic levels of the inflammatory cytokines TNF-α, IL-1β and IL6, as well as the inhibition of MMP-2 and -9^30,31^ that are elevated in limb muscles and spinal cord from hSOD1G93A ALS models and patients^58^, this protecting motor neurons and modulating inflammation^32,33^.

Violacein control of the levels and signal locations of TNF-α, IL-1β and IL6 may impact not only in their roles as direct inflammatory effectors but also when acting as bridges between different pathological mechanisms. In this sense, it may interfere with the processes in which TNF-α links inflammation and excitotoxicity^59^, or IL-1β accelerates disease progression^60^ or  IL6 spreads inflammation into endothelial cells^61^. Therefore,violacein could modulate/inhibit multiplying damage effectors and targets in ALS.

Treatment done also evidenced that violacein seemed to impair the emergence of glial reactive phenotypes that are deleterious to neurons^34^ and that release pro-inflammatory molecules that activate damaging CNS cascades, thus constituting a positive feedback loop that may account for progressive neurodegeneration^7,34,59–61^. This effect could be linked to the prevention of glial reactivity ( astrocytosis and microgliosis) in Tg-treated animals, including the capacity to abolish the deleterious actions of reactive glia or to preserve the microglia-astrocyte communication that is necessary for proper CNS function^6–12^. Thus, in the ALS model used, violacein may had interfere the vicious feedback between inflammation and dysfunctional glial cells, repressing its preponderant deleterious effects during disease onset and progression^3,4,6–8^. Moreover, in view of the peripheral effects seen upon treatment, it is possible that violacein may act on other cellular targets such as Schwann cells^8^ or the peripheral monocyte/macrophage system^51^, which appear affected in ALS, as well as on other cells,  either by direct or indirect actions that  include not only the reported here but also by  impeding (or repairing) disease-disturbed cellular communication^7,8,50^. Data obtained also reinforces the concept that the modulation of the emergence and proliferation of aberrant glial cells could mitigate ALS effects^16,51^. In summary, evidence presented here indicates that violacein could exhibit the potential pharmacological efficacy to prevent some ALS devastating effects, and that it deserves further study, mainly in view of the few therapeutic approaches available for this yet incurable disease.

## Supplementary Information

Supplementary Information.

## Data Availability

Datasets generated during the current study are available from the corresponding author on reasonable request.
