# Supplementary material for: Neuroprotective effects of violacein in a model of inherited amyotrophic lateral sclerosis
Source: Sci Rep. 2022 Mar 15;12:4439. doi: 10.1038/s41598-022-06470-7 (PMC8924276; doi:10.1038/s41598-022-06470-7)

Neuroprotective effects of violacein in a model of inherited amyotrophic lateral sclerosis

Silvia Olivera-Bravo[1],*, Carmen Bolatto[1,2], Gabriel Otero Damianovich[1], Matías Stancov[1], Sofía Cerri[1,2], Paola Rodríguez[1], Daniela Boragno[1], Karina Hernández Mir[2], María Noel Cuitiño[1], Fernanda Larrambembere[1], Eugenia Isasi[1,2], Diego Alem[3], Lucía Canclini[3], Marta Marco[1,4], Danilo Davyt[5], Pablo Díaz-Amarilla[1,6].

[1] Cell and Mol Neurobiol Lab, NCIC Department, Instituto de Investigaciones Biológicas Clemente Estable (IIBCE), Montevideo, Uruguay

[2] Histology and Embryology Department, Faculty of Medicine, Universidad de la República (UdelaR), Montevideo, Uruguay

[3] Genetic Department, IIBCE, Montevideo, Uruguay

[4] Tumoral Biol Area, Clin Biochem Department, Faculty of Chemistry, UdelaR, Montevideo, Uruguay

[5] Pharm Chem Lab, Organic Chemistry Department, Faculty of Chemistry, UdelaR, Montevideo, Uruguay

[6] Deceased author

* Corresponding author, solivera@iibce.edu.uy

Supplementary information

Upper left: Supplementary figure 1 (Fig. S1)

Fig. S1 AbAs morphological features. (**a**) On the left appears a representative light image of the mixed glial culture obtained from the lumbar spinal cord of a 200 day-old Tg rat at 5 days in vitro showing microglial cells (black arrows) and elongated cells that resemble astrocytes (white arrows). On the right appears an AbAs culture representative of the cells used in this work. Upon 6 consecutive passages, each one done every 5 days, the initial mixed glial culture shown in the left picture will result in AbAs, with the elongated shape as the predominant, almost exclusive phenotype, without microglial cells in the entire field imaged. (**b**) Confocal images of the mixed glial culture (left) and p6 AbAs (right) once labelled with S100β (green) that recognizes astrocytic phenotype (green arrows) and Iba1 (red) that identifies microglial cells (red arrows). The co-existence of both cellular types in the glial mixed culture disappeared upon successive passages causing that microglial cells were hardly detected cultured AbAs. (**c**) p6 AbAs exposed to vehicle (0.25% DMSO, left) or to 100 nM violacein (right) during 24 h and then labelled with GFAP (red) and Alexa 488 Phalloidin (green). The diffuse GFAP signal reported as a characteristic AbAs feature[11,14] almost disappeared upon violacein treatment. Note the simpler pattern of phalloidin labelling likely due to the significant cell death as indicated by decreased density of Hoechst 33342 positive cell nuclei (light blue) in AbAs incubated with violacein. (**d**) Confocal images of p1 cultured adult astrocytes exposed to vehicle (left) or 100 nM violacein (right) during 24 h and then immunolabeled against GFAP (green) and S100β (red). Note the preserved cellular density upon violacein treatment and the tendency to have more cells positive to S100β. Cell nuclei were labelled with Hoechst 33342. Calibration: 75 µm (**a**); 50 µm (**b** and **c**); 30 µm (**d**).

Upper right: Entire original gels of the muscle zymography used in Fig. 5c

Scannings of whole zymograms from the homogenates of soleus muscles from each experimental

condition. This approach allowed recognizing the activities of MMP-9 (upper white band), MMP-2 (mid white band) and degraded MMP-2 (bottom white band) in violacein-untreated (Untreated, upper gel) and –treated samples (Treated, bottom gel). Lanes included in Fig. 5c appear inside the red rectangles. The positive control composed by recombinant MMP-2 and MMP-9 appear in separate lanes under the title of rMMPs. Other unnamed lanes were not employed and were complementary internal controls (lanes on the left) or other experimental conditions. Gel scanning was done with an iBright FL1500 (Thermo Fisher) reader.

Bottom left: Original dot blots from Fig. 6a

Inverted and right images of the original dot blots corresponding to TNF-α, IL-1β and IL-6 signals (upper, mid and bottom pairs of pictures) of each experimental condition. The same amount of protein was used in each condition. Membrane scanning was done in the iBright FL1500 reader.

Upper right: Entire original gels of the spinal cord zymography employed in Fig. 8c

Scanned whole gel zymograms of the spinal cord homogenates from each experimental condition, appearing violacein untreated and treated samples in the upper and bottom gel, respectively. Lanes included in the figure appear inside the red rectangles. Controls and gel scanning was done as in Fig. 5c.

Fig. S1

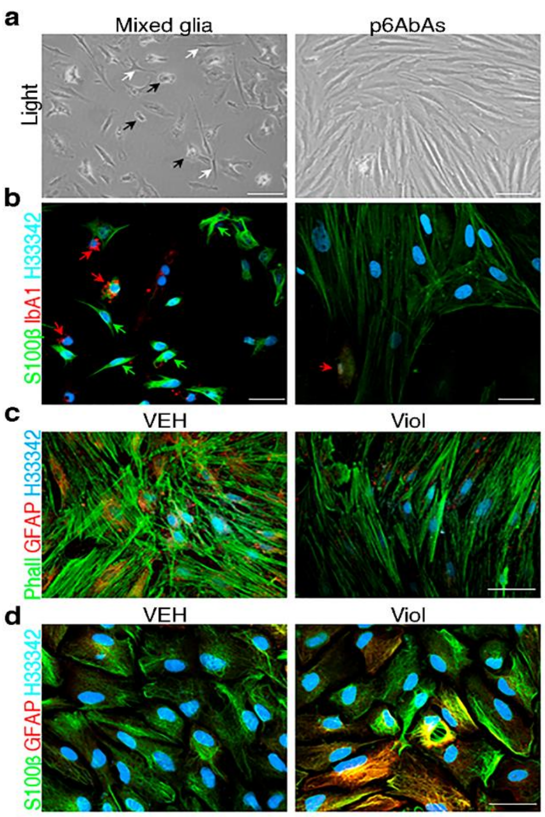

Fig. 5c

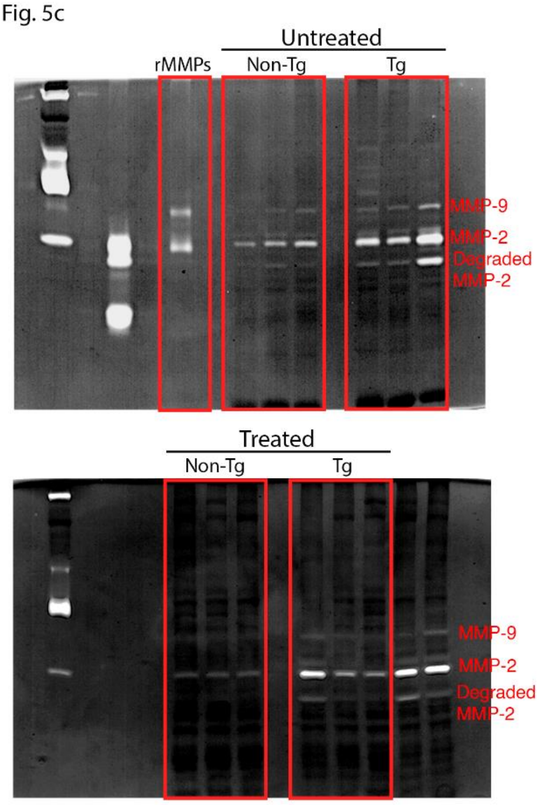

Fig. 6a

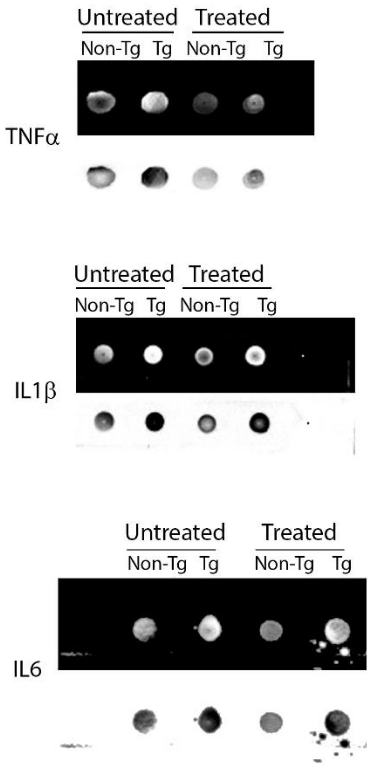

Fig. 8a

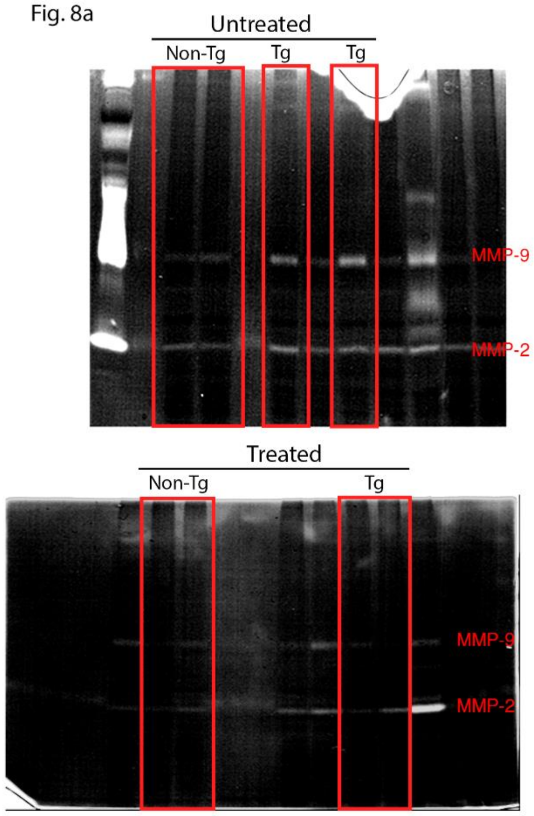

Supplement: Supplementary file 1 — Supplementary Information. [file 41598_2022_6470_MOESM1_ESM.pdf]
